# Flat Feet, Happy Feet? Comparison of the Dynamic Plantar Pressure Distribution and Static Medial Foot Geometry between Malawian and Dutch Adults

**DOI:** 10.1371/journal.pone.0057209

**Published:** 2013-02-28

**Authors:** Niki M. Stolwijk, Jacques Duysens, Jan Willem K. Louwerens, Yvonne HM. van de Ven, Noël LW. Keijsers

**Affiliations:** 1 Research Department, Sint Maartenskliniek, Nijmegen, The Netherlands; 2 Department of Orthopaedics, Sint Maartenskliniek, Nijmegen, the Netherlands; 3 Research Center for Movement Control and Neuroplasticity, Department of Biomedical Kinesiology, Katholieke Universiteit Leuven, Leuven, Belgium; Universidad Europea de Madrid, Spain

## Abstract

In contrast to western countries, foot complaints are rare in Africa. This is remarkable, as many African adults walk many hours each day, often barefoot or with worn-out shoes. The reason why Africans can withstand such loading without developing foot complaints might be related to the way the foot is loaded. Therefore, static foot geometry and dynamic plantar pressure distribution of 77 adults from Malawi were compared to 77 adults from the Netherlands. None of the subjects had a history of foot complaints. The plantar pressure pattern as well as the Arch Index (AI) and the trajectory of the center of pressure during the stance phase were calculated and compared between both groups. Standardized pictures were taken from the feet to assess the height of the Medial Longitudinal Arch (MLA). We found that Malawian adults: (1) loaded the midfoot for a longer and the forefoot for a shorter period during roll off, (2) had significantly lower plantar pressures under the heel and a part of the forefoot, and (3) had a larger AI and a lower MLA compared to the Dutch. These findings demonstrate that differences in static foot geometry, foot loading, and roll off technique exist between the two groups. The advantage of the foot loading pattern as shown by the Malawian group is that the plantar pressure is distributed more equally over the foot. This might prevent foot complaints.

## Introduction

Although many people experience foot problems in the west [1], [2] foot problems seem to be rare in Africa [3], [4]. This might be due to the shoe wearing habits, the foot shape and/or the loading pattern. While the influence of shoes on the foot is currently at the focus of interest in footwear science [5]–[8], the role of the foot shape and especially the accompanying loading pattern is yet underexposed. Therefore the aim of this study was to investigate the difference in foot loading between shod African and Caucasian adults.

Already in 1905, sir Phil Hoffman demonstrated irreversible damage to the forefoot due to wearing shoes. He stated: ‘because of the tightness of the "modern" shoe, the habitual wide shape of the forefoot, with lots of space between the toes (necessary for grasping functions) is lost [9]’. Although the design of the shoes is modernized during the years, more recent literature confirms Hoffman's idea; common foot problems, such as forefoot pain and hallux valgus, are related to the use of (inadequate) footwear in daily life [10]–[13]. Furthermore, studies have shown that there are differences between shod and unshod groups in foot biomechanics, for instance in plantar pressure distribution [5] or height of the MLA [5], [14], [15]. For example D'Aout and colleagues demonstrated that the unshod Indian adults had more loading under the midfoot area compared to the habitual shod Indian and a western group, indicating a lower MLA for the unshod groups [5].

However, the difference in biomechanical function of the foot between groups of different ethnic descents can not only be a result of shoes. Dunn et al. [16] reported that flat feet are more common in shod African Americans compared to shod non-Hispanics white and shod Puerto Ricans [16]. However, they used a fairly uncommon method to assess the foot structure; a participant was considered to have flatfeet if the examiner was unable to insert his/her fingers under the arch of the foot with the participant in a standing position [16]. Nevertheless, a more reliable study of Igbigbi et al. [17] confirmed the results of Dunn et al. [16], as they measured the plantar pressure dynamically using a blue print and found that the AI was statistically higher in Malawians (indicating a lower medial longitudinal foot arch) compared to Caucasian-Americans [17]. Also the recent study of D'Aout revealed ethnical differences, as the midfoot area of both the shod and unshod Indians was significantly more loaded compared to the western population, indicating that the Indian group as a whole had flatter feet compared to the western group [5].

The more equal distribution of plantar pressure found in the African and Asian population groups might be a preventive factor for foot complaints, as the development of foot complaints is often associated with overloading of the (fore)foot [18]–[21]. Data at pixel level of dynamic plantar pressure measures and, probably more important, on the loading pattern (roll off) of the feet of such an habitual shod African group is, however, still missing. Therefore, the aim of this study was to compare the static foot geometry, dynamic plantar pressure pattern and roll off of the foot (at pixel level) between Malawian and Dutch shod adults without a history of foot complaints.

## Materials and Methods

### Subjects

Seventy-seven subjects in Malawi and 77 subjects in the Netherlands participated in this study. The Malawian subjects (25 males and 52 females, aged between 19 and 60 years), were employees of Queens Elizabeth Hospital in Blantyre or were guardians of patients treated in this hospital. All lived in Blantyre or its surrounding areas. Of all the Dutch subjects (29 males and 48 females aged between 19 and 59 years), 37 subjects were employees of the Sint Maartenskliniek (SMK) or acquaintances of the researchers (SMK group) and 40 subjects were participants of a local long distance march (Nijmegen group). The SMK and Nijmegen group were similar in terms of activity level (active but not highly trained), BMI (24,6) and plantar pressure distribution. All subjects were accustomed to normal daily use of shoes, had no history of foot problems and were free from orthopaedic or neurological problems that could affect the walking pattern. The institutional review board approved the study. All Dutch subjects signed informed consent. As many Malawian subjects were unable to read and/or write; the subjects of the Malawi group gave their spoken consent, which was documented in a data sheet.

Three questions about the shoe/walking habits of a person were asked: 1) whether they generally worn shoes or preferred to walk barefoot, 2) what kind of shoes they used, 3) how many hours they generally walk each day.

### Measuring equipment and protocols

#### Plantar pressure

In Malawi, plantar pressure data were collected using a Footscan® USB plate. In the Netherlands, a Footscan® 3D plate (RSscan, Olen, Belgium) mounted on top of a force plate (Kistler, Winterthur, Switzerland) was used. Both plates have the same active sensor surface (0.48m in length and 0.32m in width) and spatial resolution (2.6 sensors/cm^2^). All participants walked barefoot over the pressure plate at their preferred walking speed. Participants walked according to the three-step protocol (the third step was measured), alternating with the left and right foot. This action was repeated until six steps were obtained (three recordings of each foot). A trial was repeated if the entire footprint was not recorded or an irregular walking pattern was observed.

The data were collected at 300 Hz for the Malawi group, 200 Hz for the SMK group, and 500 Hz for the Nijmegen group. The reason why different measurement frequencies were used for the three groups, is the use of different measurement systems. In case of the SMK group, the plantar pressure measurement was combined with a walking speed recording using a VICON motion analysis system. This was only possible in our laboratory at 200 Hz. In case of the Nijmegen group and the Malawi group the pressure plate was not connected to the VICON system and therefore the maximum possible measurement frequency of each system was used: 500 Hz for the 3D system and 300 Hz for the USB system.

For the SMK group, walking speed was measured using the VICON system by placing two markers on each foot; one on the distal part of the second metatarsal bone and one on the heel. The walking speed was calculated by dividing the distance between two heel strikes of the same foot by the time required to cover that distance. The Pearson correlation test was used to test the relationship between walking speed as measured with VICON and the contact time on the pressure plate. As for the remaining 40 Dutch subjects and the Malawi group only contact time was measured. Contact time was used to test for statistical differences in speed between the Malawian and the Dutch group using an unpaired t-test.

#### Static foot geometry

**Figure 1:**
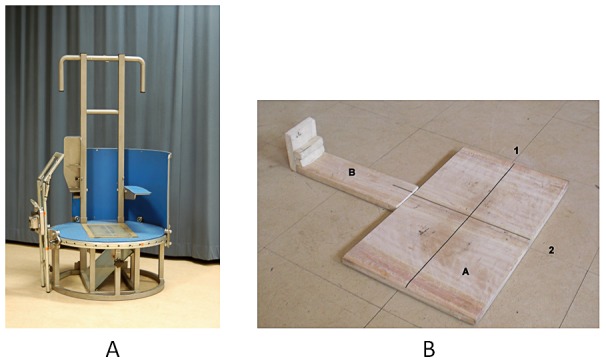
Measuring equipment used in the Netherlands (**Figure 1A**) and in Malawi (**Figure 1B**). Abbreviations used in Figure 1B: A = board for placement of the feet, B = board for placement of the camera,1 = longitudinal line, 2 = mediolateral line

Static foot geometry was measured using two different measuring instruments. In the Netherlands the geometry was measured using the Foot Build Registration System (FBRS), as described by Tuinhout et al. [22] (see Figure 1A). The subjects were standing in upright position, with one foot on the platform and the contralateral foot placed on a higher support and were able to maintain balance by holding a bar in front of them. The ankle of the examined foot was placed in 90 degrees with extended knee. The subjects were asked to fully weight bear the examined leg. On the platform one longitudinal line and 39 vertical lines were marked. The center line of the 39 vertical lines (0-line) will be referred to as the mediolateral line. A Canon PowerShot A530 digital camera was attached to a moveable frame enabling pictures to be taken from standardized directions.

In Malawi a measuring instrument was designed (see Figure 1B) based on the FBRS. This measuring instrument consisted of a platform board (0.60m by 0.30m) and a slat (0.20m by 0.10m). On the platform board, a longitudinal and a mediolateral line were drawn similar to the longitudinal and mediolateral line on the FBRS. The slat was used as a support to place the digital camera in a standard way. The subjects were able to maintain balance by holding the back of a chair which was placed directly in front of them. As a higher support for the contralateral foot was missing, we asked the subjects to lift their contralateral foot instead.

Before registration, five anatomical landmarks were marked by using a kohl pencil. The anatomical landmarks included: the center of the medial malleoli, the midpoint of the tuberculum of the os naviculare, the medial center of the distal and the proximal head of the first metatarsal bone and the distal medial point of the calcaneus [23]. Static foot geometry was measured under 1 body weight load by taking a digital picture of the medial side of the foot during full weight bearing. The foot was positioned in such way that the second metatarsal head and the dorsal calcaneus landmark was in line with longitudinal line on the platform board in Malawi and the Netherlands. Furthermore, the navicular landmark was positioned at the mediolateral line in Malawi and the Netherlands. Pictures of both feet were taken of each subject.

#### Reliability of the systems

The FBRS system, as used in the Netherlands, has a good/sufficient reproducibility and reliability [22]. Although the design of the instrument in Malawi was based on the FBRS (similar distance between the foot and camera), the reliability of the system was unknown and was investigated. For this purpose, two subjects were measured several times. First, the reliability of the camera placement was tested by taking four pictures of the medial site of each foot of the two subjects when standing still on the platform. After each picture, the camera and the slat were removed and returned in the same position. Secondly, the reliability of the placement of the foot was determined in a similar manner. For this measurement, the camera position remained unchanged, but after each picture the foot was lifted from the board and repositioned. Reliability was calculated with the Kendall's W nonparametric test for correlation.

### Analysis

Either the left or the right foot of each subject was included in the analysis. This foot was randomly selected.

#### Plantar pressure

First, the mean pressure per sensor (MP), peak pressure per sensor (PP) and pressure-time integral per sensor (PTI) were calculated for each step per foot for each subject. Subsequently, plantar pressure data were normalized for foot size, width and foot progression angle according to the method developed by Keijsers et al. [24]. For each subject and for each foot, the mean MP, PP and PTI over the trials were computed. To eliminate the effect of body weight and walking velocity on the plantar pressure distribution, the plantar pressure was normalized for the total pressure under the foot.

To deal with the large number of sensors in statistical analysis, we used the procedure described previously by Stolwijk et al ([25]). This technique involves a nonparametric procedure, based on grouping all adjacent sensors that exhibit similar difference in sign (an increase or decrease in MP, PP or PTI). In the present study the number of groups for sensors was 7. Therefore, the p-value was adjusted by using the general Bonferroni correction (α/N) in advance, in which the N was the number of sensor groups (7). Hence, the level of significance for the plantar pressure distribution in this study was set at 0.007.

To check whether the measured data of the two pressure plates (3D and the USB) were comparable, additional data of 20 subjects of the SMK group were recorded. For this purpose, the USB plate (used in Malawi) was put directly behind the 3D plate (used in the Netherlands). Subsequently, all 20 subjects walked ten times at their preferred walking speed over both plates. Differences in measured MP between the plates were tested with a paired t-test, using the same analysis technique as explained above.

In addition, the trajectory of the Center of Pressure (CoP) was calculated for each subject per step using the normalization method of Keijsers et al. [24] and described before by Stolwijk et al.[26]. The mean CoP trajectory for each subject was normalized for the duration of the stance phase (0–100%). In addition, the velocity of the CoP (vCoP) (the derivative of the CoP trajectory in anterior-posterior direction) was calculated to investigate temporal differences. To be able to compare the vCoP between both groups, the CoP was normalized for the mean vCoP of each group (a relative VCoP).For each percent of the stance phase, the CoP position in the mediolateral direction (x-direction) and anterioposterior direction (y-direction) and the vCoP of the Malawian group was compared to the Dutch group with an unpaired t-test. For this analysis, the alpha was set at 0.0005 to correct for the amount of tests performed (100).

Arch Index (AI), was calculated to quantify foot geometry based on plantar pressure data. The AI was calculated as described by Cavanagh and Rodgers [27] using the footprint of the MP of each subject. They indicated the arch as low arch (AI≥0.26), normal arch (0.21<AI<0.26) or high arch (AI≤0.21). Furthermore, the ratio foot width/foot length was calculated based on the raw plantar pressure data of each subject. For this calculation, the foot length was defined as the length of the foot from the proximal point of the heel and the distal point of the forefoot of the contour line of 10 kPa, so it did not involve the toes. The foot width was defined as the horizontal distance between the most medial and lateral point of the contour line of 10 kPa for the forefoot area. Difference in AI and foot width/foot length ratio between the Dutch and Malawian group was tested with an unpaired t-test. Furthermore, association between the descent (Dutch or Malawian) and the AI category (low, normal or high) was tested using a chi^2^ test with Cramer's V statistics. For all tests the α was set at 0.05.

#### Static foot geometry

**Figure 2:**
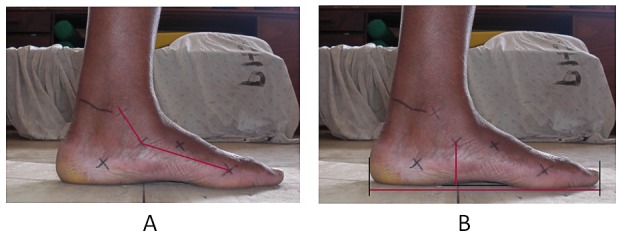
Static foot geometry. Figure 2A: Medial Angle. Angle between the center of the medial malleolus, the navicular tuberculum and the medial center of the first distal metatarsal head. Figure 2B: Navicular height/foot length ratio.

The same foot as was used for the plantar pressure analysis was used for the analysis of the static foot geometry data. All collected pictures were downloaded to a computer. Using a custom written Matlab® R2006a for Windows (The MathWorks, Inc.) program, the anatomical landmarks were marked on the screen. The medial angle was defined as the angle between the center of the medial malleolus, os naviculare, and the medial center of the distal head of the first metatarsal bone (see Figure 2A). Also the NF ratio was calculated (see Figure 2B). The foot length was defined as the length between the most distal point of the heel and the most proximal point of the big toe. The navicular height was the perpendicular distance between the os naviculare and a line between the points where the anterior and posterior part of the MLA first touched the platform. Statistical difference in medial angle and NF ratio between both group were tested by means of an unpaired t-test with an α of <0.05.

#### Regression Analysis

In principle, differences in plantar pressure between the Malawian and Dutch group could be the result of covariates such as body weight and walking velocity. Therefore, a stepwise multiple regression analysis with forward selection was performed to find the set of predictors/variables that were most effective in predicting the MP at each sensor. The independent variables were: group (Malawian or Dutch), weight, age, gender, AI, contact time, foot length, medial angle, NF ratio and foot length/foot width ratio. To be able to compare the regression coefficients between the different independent variables, the input parameters were normalized between 0 and 1.0. Because of the large number of sensors, we present: 1) the percentage of sensors in which an independent variable was included in the regression model, 2) the parameters which were most often selected as the most important parameter in the regression model

## Results

### Subject characteristics

The characteristics of the subjects of the Malawian and the Dutch group are given in Table 1. Sixty of the 77 Malawian subjects (75%) walked on normal solid shoes, 6 on sport shoes, 7 on flip flops, 3 on high heels and 1 on crocs. All indicated to wear shoes most of the time and walked on average 3.1 (SD 2.1) hours per day. All subjects of the Dutch group used shoes every day and indicated to wear different shoes during the week, but mostly normal solid shoes. On average the subject of the Dutch group walked 2.5 (SD 2.1) hours per day. All subjects could perform the heel rise test and showed normal flexibility and normal supinatory potential of the foot.

**Table 1:** Subject characteristics.

	Malawi (N = 77) (*Mean (SD))*	The Netherlands (N = 77) (*Mean (SD))*
Age (years)	37.63 (11.44)	40.08 (10.17)
Weight (kg)	63.23 (10.03)	75.70 (13.45)
Male (count)	25	29
Female (count)	52	48

Eight of the 77 pictures of the Malawian subjects could not be used because the longitudinal line was not visible or the lower leg was clearly not situated above the foot. Because of this, two Malawian groups were used: one for the plantar pressure data (77 people) and one to quantify the static foot geometry (69 people).

### Plantar pressure

#### Speed

A significant difference (p<0.0001) in contact time between the Malawian and the Dutch group was found; the Malawian group had a mean contact time of 0.80 seconds (0.09) and the Dutch group 0.66 seconds (0.05). For 20 subjects of the Dutch group, contact time and speed was measured simultaneously. From these data a correlation coefficient between contact time and speed of -0.65 was found. Using the accompanying linear regression equation for the line of best fit: y = −2.7x+3.25 (in which y = speed and x = contact time), the overall walking speed for the Dutch group was estimated to be 1.25 m/sec, while it was 1.1 m/sec for the Malawi group.

#### Mean pressure, peak pressure and pressure-time integral

The correlation coefficient between the MP and the PP, and the correlation coefficient between the MP and the PTI was calculated for each sensor. The correlation coefficient was 0.92 (SD 0.06) between MP and the PP, and 0.95 (0.06) between the MP and the PTI for both groups. Based on these findings, it can be said that the MP, PP and PTI in both groups were distributed in a similar matter, which is in accordance with previous findings on this topic [28], [29] Therefore, we chose to show the results of the MP as representative for the PP and the PTI. Furthermore, there was no significant difference in MP at any pixel between the measurements done with the USB and the 3D plate for the 20 subjects who walked over the USB and the 3D plate in the same trial. Therefore, it can be concluded that there was no significant difference in measured plantar pressure between the pressure plates.

**Figure 3:**
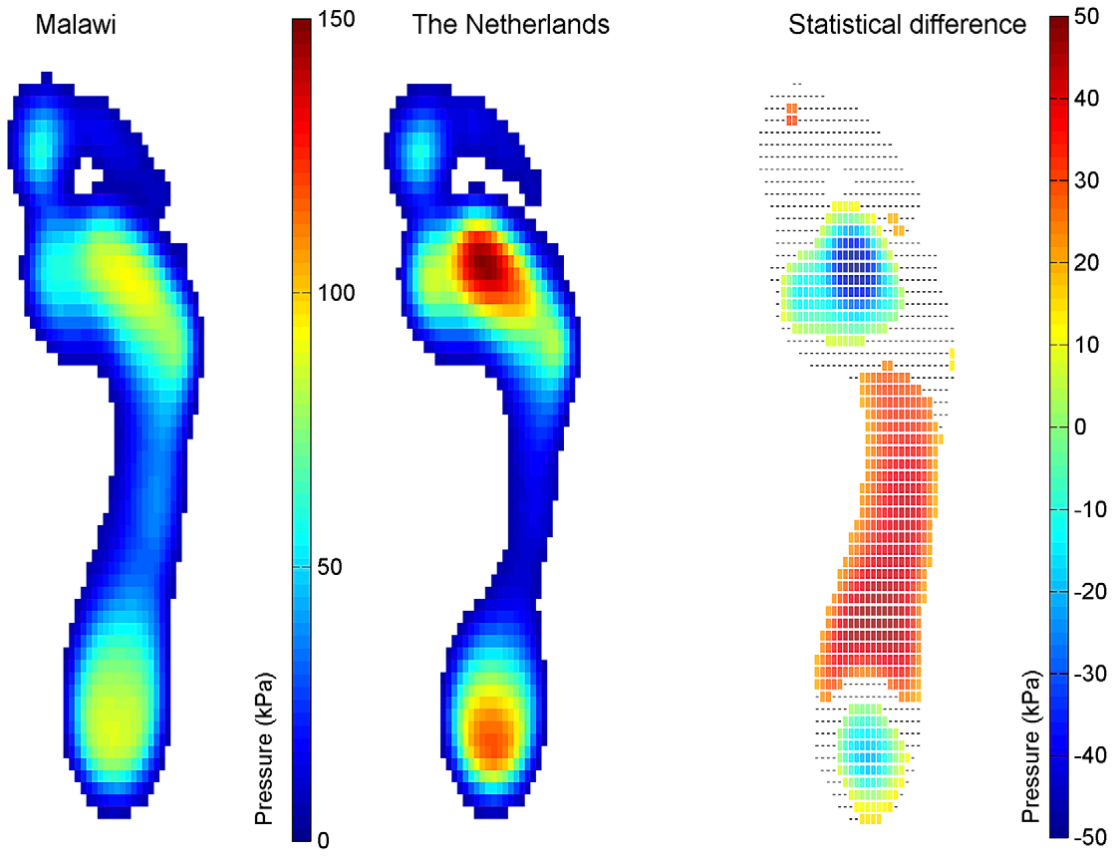
Mean plantar pressure. Left panel: The MP distribution for the Malawian group; middle panel: The MP distribution for the Dutch group; Right panel: The difference in MP between the Malawian and Dutch group. The coloured squares indicate that the MP is statistically different (p<0.007) between the groups and the black small lines indicate that the groups were not significantly different. Note that for both groups only pixels are shown with a mean output above 0.5N

The left and middle part of Figure 3 shows the mean MP for the Malawian and the Dutch group. Statistical analysis revealed that the MP is significantly (p<0.007) larger under the midfoot and lower under the heel and the metatarsal head II and III (see right part of Figure 3) for the Malawian group.

**Figure 4:**
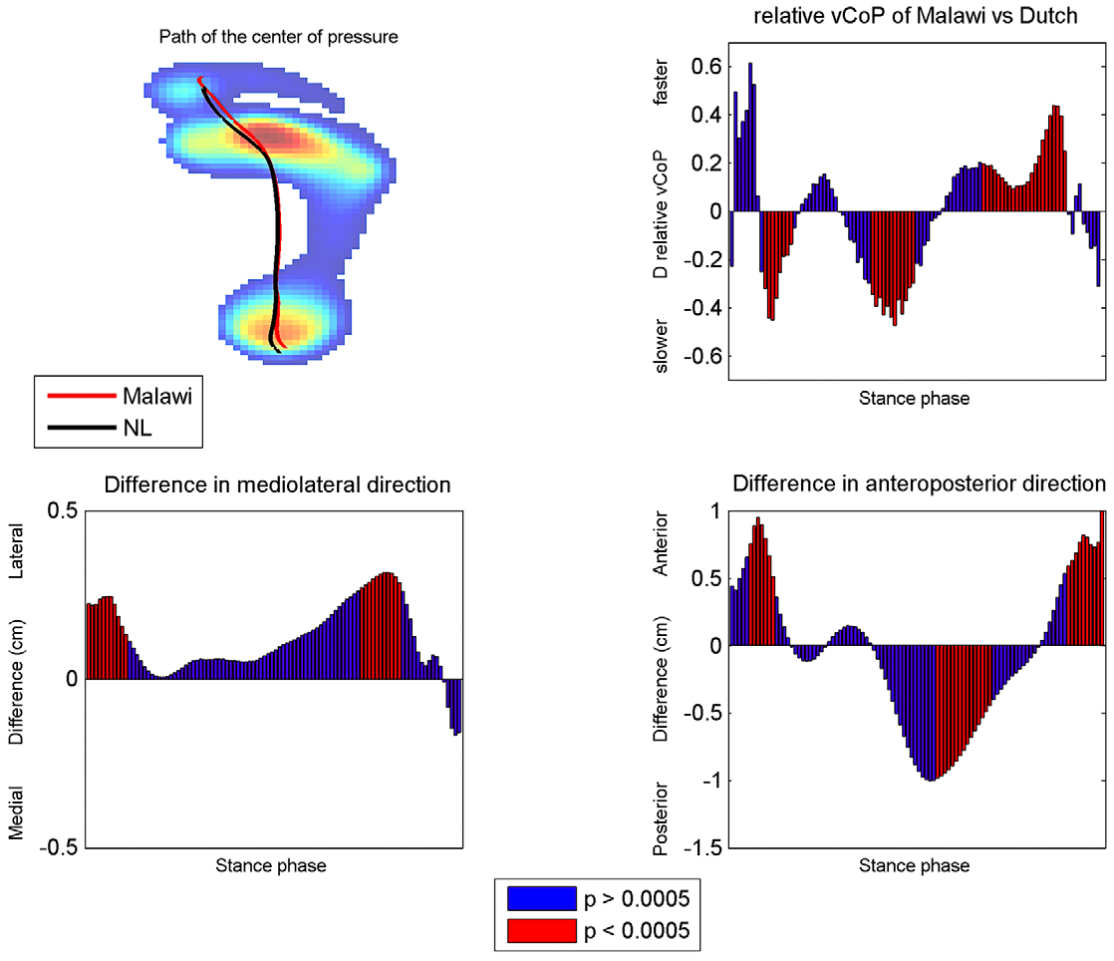
Trajectory of the Center of Pressure. Upper left panel: the MP distribution for the Dutch group including the CoP path of the Dutch and Malawian group. Upper right panel: Difference in relative vCoP: Malawi group minus Dutch. Lower panels: the difference in CoP path for the mediolateral (left panel) and anteroposterior (right panel) direction. The red bars indicate that the CoP path/vCoP differs significantly between both groups.

The difference in CoP position and vCoP between the Malawian and Dutch group during roll off is shown in Figure 4. The Malawian subjects roll off their feet more laterally (a positive difference indicates lateralization of the CoP) during most part of the stance phase. In the first (1–14%) and third part (62–87%) of the stance phase, this difference between the Malawian and Dutch group was significant(p<0.0005). For the anteroposterior direction, the CoP was located significantly more anterior just after heel strike (6–12%) and before toe off (91–100%) for the Malawian subjects. In contrast, the CoP was situated significantly more posterior during mid stance (56–70%). The relative vCoP was significantly higher after heel strike and during propulsion and lower during mid stance for the Malawi group.

#### Foot width/foot length ratio

The Malawian feet had a significant higher foot width/length ratio (0.46 (0.03)) compared to the Dutch feet (0.44 (0.02)) p<0.001. This is most likely due to the difference in foot length, which was significantly different between both groups (p<0.0001), whereas foot width was not significantly different between both groups (p = 0.28).

### Static foot geometry

#### Reliability Malawian measuring instrument

The reliability of the camera and foot placement of the Malawian measurement equipment was good to excellent. For the placement of the camera a Kendall's W of 0.925 for the medial angle and also a Kendall's W of 0.925 for the NF ratio was found. For the placement of the foot, we found a Kendall's W of 0.911 and 0.778 for the medial angle and the navicular height/foot length ratio, respectively.

#### Medial angle and navicular height/foot length ratio

The Malawian foot was significantly different from the Dutch for the medial angle (p = 0.046), the NF ratio (p<0.001) and navicular height (p<0.001). The Malawian group had a smaller medial angle (139.69 (9.47)°) and navicular height/foot length ratio (0.17 (0.03)) compared to the Dutch group (144.05 (8.83)° and (0.20 (0.03) mm) respectively) indicating a lower media arch. In contrast to the foot length based on the plantar pressure measurements, there was no significant difference in foot length between both groups (p = 0.13), when measured on the picture. All results are given in Table 2.

**Table 2:** Foot geometry.

	Malawi (Mean(SD))	The Netherlands (Mean(SD))
Medial angle (°)*	139.69 (9.47)	144.05 (8.83)
Ratio navicular height/foot length*	0.17 (0.03)	0.20 (0.03)
Navicular height (mm)*	44.88 (6.56)	52.76 (7.87)
Foot length (mm)	266.02 (15.77)	270.22 (17.59)
Foot width/length ratio*	0.46 (0.03)	0.44 (0.02)
AI*	0.28 (0.03)	0.21 (0.06)
Low arch (%)	76.6	26.0
Normal arch (%)	22.1	33.8
High arch (%)	1.3	40.3

* significantly different between the Malawi and Dutch group at p<0.05

#### Arch index

A statistically significant difference was measured for the AI. The Malawian group had a mean AI of 0.28 (0.03), whereas the Dutch group had a mean AI of 0.21 (0.06). The Malawian group had most subjects (76.6%) in the low arch group, whereas the Dutch group had most subjects in the high arch group (40.3%) (see Table 2). There is a significant (p = 0.00) Cramers V association of 0.566 between the AI classification (low, normal of high) and the descent (Malawian or Dutch).

### Regression analysis

A stepwise multiple regression analysis revealed that AI, group and NF ratio were chosen as a predictor of plantar pressure for the majority of the sensors and almost always chosen as the first parameter. The AI was selected as a predictor for 60% of the sensors and was selected as the most important predictor in the regression equation for 42% of the sensors. Group was selected as a predictor for MP for 38% of the sensors and was selected as the most important predictor for 23% of the sensors. NF ratio was included in the regression equation for 37% of the sensors and was the first predictor of the MP per sensor for 9% of the sensors. All remaining parameters contributed for less than 30% in the prediction of MP (weight: 21%, age: 13%, gender: 15%, contact time: 29%, foot length: 26%, medial angle: 18%, foot length/foot width ratio: 18% of the sensors).

**Figure 5:**
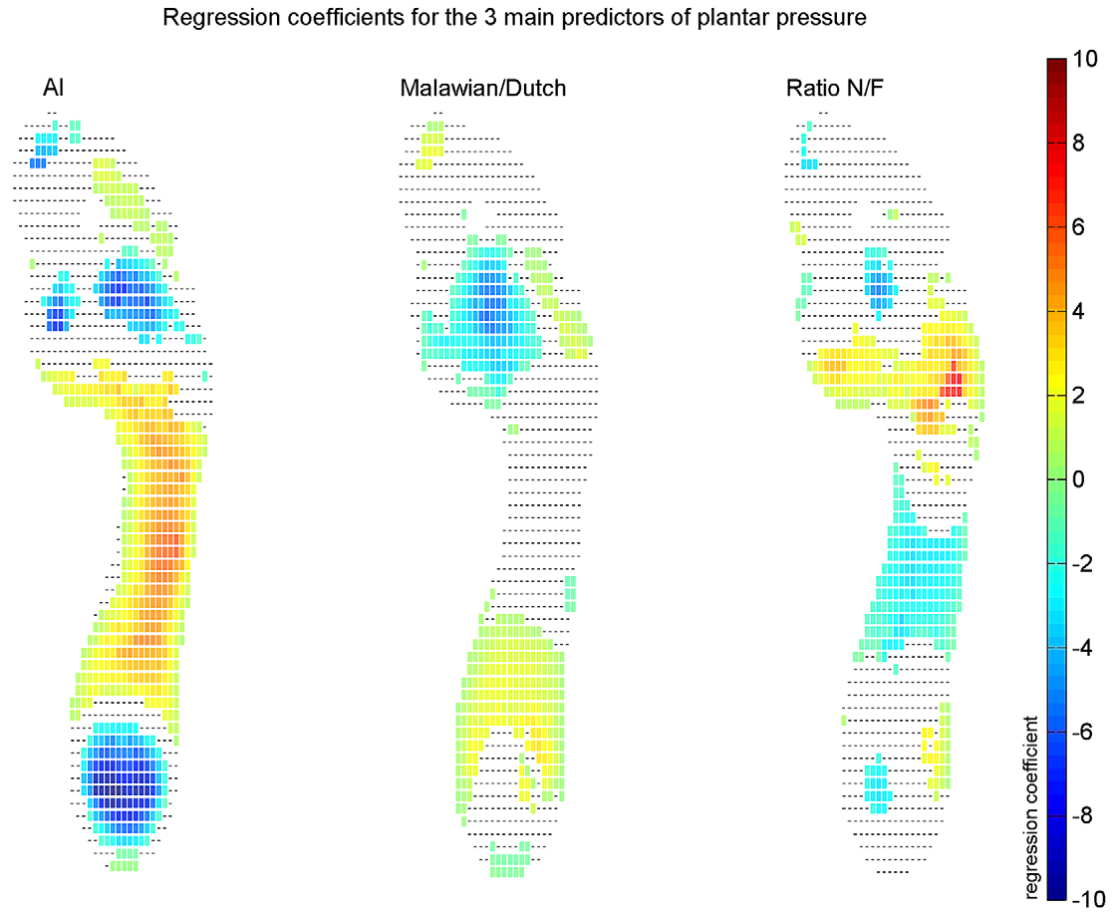
Regression coefficients for each sensor for the 3 most important predictors: AI (left panel), group (middle panel) and NF ratio (right panel). The coloured squares represent the regression coefficients for the sensors for which the parameter was an predictor.

Figure 5 show the regression coefficients for the AI, group and NF ratio for the sensors in which the variable was selected in the regression model. The regression coefficients were positive at the mid foot region and negative at the heel and forefoot region for the AI, indicating that a higher AI (flatter MLA) causes more pressure under the mid foot and less pressure under the heel and forefoot. The coefficients for group were positive for the sensors at the proximal mid foot area and negative for the forefoot sensors, indicating that being a Malawian adult cause less pressure under the forefoot and more pressure under the proximal part of the mid foot. The regression coefficients for the NF ratio are positive for the proximal part of the forefoot and negative for the mid foot, which indicates that a higher NF ratio (a higher MLA) cause less pressure under the mid foot and more pressure under the proximal part of the forefoot

## Discussion

This study is the first to describe the dynamic foot loading at pixel level of a large group of African subjects. Major differences between the Malawian and Dutch group were identified in foot loading. The Malawian subjects had a significant flatter MLA, more loading under the midfoot and a shorter period of forefoot loading during the roll off. All these findings might explain why people of African descent experience less foot problems compared to their Caucasian/European peers.

The present study showed that the MLA is flatter for the Malawian group and demonstrated that this group had a larger loading area. Consequently, the plantar pressure is distributed more equally over the foot. Unequal foot loading, i.e. the local peak pressures under the forefoot, have been related to the development of metatarsalgia [18] and fasciitis plantaris [21]. The larger midfoot loading is consistent with the results of Igbigbi et al. [17] who identified a difference in AI between Malawians (0.26 (0.07)), white Americans (0.23 (0.05)) and Europeans (0.23 (0.05)).

One of the most novel finding of this study is the significant different roll off pattern of the Malawi group compared to that of the Dutch subjects. The position of the CoP of the Malawians was anterior from the CoP position of the Dutch during both the heel strike and the propulsion phase of gait and posterior during midstance (Figure 4). Furthermore, the vCoP was significantly slower at heel strike and mid stance and faster at propulsion for the Malawi group. This difference in the velocity and trajectory of the CoP probably reflects a roll off pattern with a landing on a more distal part of the heel, a longer period of midfoot loading and a shorter period of forefoot loading with propulsion at a more distal part of the toes. Especially this shorter period of forefoot loading is presumably of importance in the prevention of forefoot complaints as the forefoot is one of the most vulnerable parts of the foot and elevated forefoot loading is associated with the development of metatarsalgia (18). The observed difference in CoP location at heel strike and the contribution of the toes are similar to what was found by d'Aout et al. [5]. in unshod Indian adults and might indicate that the Malawian subjects had a flatter initial heel contact and had more involvement of the toes at propulsion [5]. Evidence of the greater contribution of the toes in flat-arched feet was also given by Levinger et al. [30] and Murley et al. [31]. They demonstated that the gait of the non-pathological low arched foot is associated with an increased plantar flexion of the toes [30] and an increased activity of the tibialis posterior and flexor hallucis longus muscle during the propulsion phase of gait [31] as compared to normal-arched subjects. Furthermore, slight differences were found for the CoP position in mediolateral direction; the CoP of the Malawian subjects was located more laterally on the foot at heel strike and towards toe off. In contrast to the found differences in CoP position in anteroposterior direction, this slight shift (max 0.3 cm) is probably not related to a difference in walking style or foot structure between the groups but related to the found difference in walking speed. Lateral foot loading is expected at slower walking speed [32], [33].

It might be argued that some of the differences in plantar pressure between Malawian and Dutch adults were due to differences in BMI or walking speed between the groups. Although data on the actual walking speed and BMI is missing, these measures are reflected in the measure of body weight, gender, foot length and contact time. It appeared that these measures are not important predictors for plantar pressure. In contrast, the AI, group and NF ratio were found to be the most important predictors of the plantar pressure. As a consequence, the distribution of pressure under the foot is largely determined by the shape of the medial column as measured by the NF ratio and AI but also by the country of origin.

The AI was found to be significantly higher for the Malawian group and an important predictor for plantar pressure. It is, however, known that the AI is related to the BMI [34], [35] and body weight. The Dutch group had a significantly larger body weight than the Malawian group. Hills et al. [34] found significantly higher peak pressures under most anatomical regions, but mostly under the mid foot, for an obese group compared to a non-obese group. Vela et al. [35] concluded that an increase in bodyweight caused an increase in plantar pressure under the first and lesser metatarsal head, midfoot, and heel regions. Unfortunately data of the body length of the Malawian group is missing and it is therefore not possible to calculate the BMI. However, in the multiple regression analysis, body length is represented in factors as gender[36] and foot length[36] and none of these factors (including body weight) were important predictors. Moreover, in our study, higher AI values were found for the lighter group (Malawi), which is contradictory to what is found in literature on the relation between AI and BMI/body weight. Hence, it is not likely that the increased mid foot pressure of the, lighter, Malawian group was due to a difference in BMI.

In Malawi a different, simpler, measurement tool was used. As the measurement equipment had to be carried from the Netherlands to Malawi and also each day to the hospital, it was necessary to use a portable pressure plate and a self-designed static foot geometry registration system. However, as both measurement systems showed to have a good reliability, it is not to be expected that the found differences in plantar pressure distribution and static foot geometry were the result of the use of different systems. Moreover, we showed that, when measuring the same person with both the two types of plates, the plantar pressure distribution was exactly the same.

Although the Malawian group was a group of shod Malawians, footwear habits and level of activity might still differ from the Dutch. Firstly, due to the temperature differences, the Malawian subject might wear open shoes or flip-flops more often compared to the Dutch. Consequently, the Malawian adults had clearly a more horny skin compared to the Dutch adults, indicating barefoot loading. Secondly, it has been shown that people in the western society overestimate their activity level [37], [38], whereas African people probably underestimate their activity level (physically active work is highly prevalent, for instance during washing, gardening, walking to and from work/market, etc). However, both groups indicated to wear "normal solid" shoes most of the day and walk approximately 3 hours per day. Overall, it is estimated that the African and European group selected were quite comparable with respect to activity level and shoe wear habits.

Based on this study we can state that there is a clear difference in dynamic foot loading and static foot geometry between the Malawian and Dutch group. The question arises whether these changes in loading are important from a clinical viewpoint. It is of interest that foot problems seem to occur less among adults of African descent. This leads us to suggest that the presently found adjustments in Malawi subjects may have a beneficial effect. We identified the following outcomes: 1) the increased loading area, 2) the more equal distribution of pressure over the foot and 3) the larger period of midfoot loading and shorter period of forefoot loading. It is striking that these characteristics fit quite nicely with some of the main current goals of the treatment of foot problems in the west, namely to pursuit an equal distribution of pressure by insoles.
